# Nupr1 Negatively Regulates Endothelial to Hematopoietic Transition in the Aorta‐Gonad‐Mesonephros Region

**DOI:** 10.1002/advs.202203813

**Published:** 2023-01-13

**Authors:** Haizhen Wang, Di Liu, Haifeng Chen, Yuqing Jiao, Haixin Zhao, Zongcheng Li, Siyuan Hou, Yanli Ni, Rong Zhang, Jinyong Wang, Jie Zhou, Bing Liu, Yu Lan

**Affiliations:** ^1^ Key Laboratory for Regenerative Medicine of Ministry of Education Institute of Hematology School of Medicine Jinan University Guangzhou Guangdong 510632 China; ^2^ Peking‐Tsinghua Center for Life Sciences Peking University Beijing 100871 China; ^3^ Chinese PLA Medical School Chinese PLA General Hospital Beijing 100853 China; ^4^ State Key Laboratory of Experimental Hematology Department of Hematology Fifth Medical Center of Chinese PLA General Hospital Beijing 100071 China; ^5^ Integrated Chinese and Western Medicine Postdoctoral Research Station Jinan University Guangzhou Guangdong 510632 China; ^6^ School of Basic Medical Sciences Southern Medical University Guangzhou Guangdong 510515 China; ^7^ Institute of Zoology of the Chinese Academy of Sciences Beijing 100101 China; ^8^ State Key Laboratory of Proteomics Academy of Military Medical Sciences Academy of Military Sciences Beijing 100071 China

**Keywords:** endothelial‐to‐hematopoietic transition, hematopoietic stem cells, hemogenic endothelial cells, Nupr1, single‐cell RNA sequencing

## Abstract

In the aorta of mid‐gestational mouse embryos, a specialized endothelial subpopulation termed hemogenic endothelial cells (HECs) develops into hematopoietic stem and progenitor cells (HSPCs), through a conserved process of endothelial‐to‐hematopoietic transition (EHT). EHT is tightly controlled by multiple intrinsic and extrinsic mechanisms. Nevertheless, the molecular regulators restraining this process remain poorly understood. Here, it is uncovered that, one of the previously identified HEC signature genes, Nupr1, negatively regulates the EHT process. *Nupr1* deletion in endothelial cells results in increased HSPC generation in the aorta‐gonad‐mesonephros region. Furthermore, single‐cell transcriptomics combined with serial functional assays reveals that loss of Nupr1 promotes the EHT process by promoting the specification of hematopoiesis‐primed functional HECs and strengthening their subsequent hematopoietic differentiation potential toward HSPCs. This study further finds that the proinflammatory cytokine, tumor necrosis factor *α* (TNF‐*α*), is significantly upregulated in Nupr1‐deficient HECs, and the use of a specific TNF‐*α* neutralizing antibody partially reduces excessive HSPC generation in the explant cultures from Nupr1‐deficient embryos. This study identifies a novel negative regulator of EHT and the findings indicate that Nupr1 is a new potential target for future hematopoietic stem cell regeneration research.

## Graphical abstract

Nupr1 deficiency resulted in increased hematopoietic stem cell generation both by promoting the specification of hemogenic endothelial cells (HECs) and strengthening their subsequent hematopoietic differentiation potential toward hematopoietic stem and progenitor cells (HSPCs). Molecularly, tumor necrosis factor α is significantly upregulated in Nupr1‐deficient HECs, and blocking which partially reduce excessive HSPC generation in Nupr1‐deficient embryos.

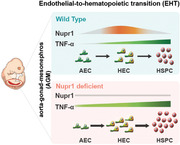

## 1 Introduction

Determining the regulatory mechanisms underlying the ontogeny of embryonic hematopoietic stem cells (HSCs) will provide new insights for in vitro HSC regeneration research. Previous work suggests that HSCs could be derived from hemogenic endothelial cells (HECs) localized in the aorta‐gonad‐mesonephros (AGM) region of mouse embryos.^[^ ^1^, ^2^, ^3^ ^]^ A subpopulation of aortic endothelial cells specifies HECs that undergo the endothelial‐to‐hematopoietic transition (EHT) to generate a relatively large number of multilineage hematopoietic progenitor cells (HPCs), and very few precursors of HSCs (pre‐HSCs) and HSCs in a short time window of embryonic development (from embryonic day (E) 9.5 to E11.5).^[^ ^4^, ^5^, ^6^, ^7^, ^8^ ^]^ Morphologically, these direct hematopoietic progenies of HECs intermingle to form intra‐aortic hematopoietic clusters (IAHCs) attached to the inner wall of the dorsal aorta.^[^ ^9^ ^]^

Canonical developmental signals, such as Notch, Fgf, and Bmp/Tgf*β* signaling pathways, as well as hematopoietic transcription factors, including Runx1, Gata2, Gfi1, and Gfi1b, are required to orchestrate the EHT process.^[^ ^1^, ^10^, ^11^ ^]^ The role of epigenetic regulation during EHT has recently been revealed.^[^ ^12^, ^13^, ^14^ ^]^ Several lines of evidence indicate that multiple inflammatory signals play a pivotal role in regulating EHT, including the tumor necrosis factor (TNF), interleukin, and toll‐like receptor (TLR) signaling pathways.^[^ ^15^ ^]^ Abrogation of certain inflammatory signals, such as blocking TNF signaling by TNF receptor gene knockout, leads to EHT blockage in vivo.^[^ ^16^ ^]^ However, the addition of certain inflammatory factors, such as interferon *γ* (IFN*γ*), could promote EHT and the production of hematopoietic stem and progenitor cell (HSPC) in an in vitro culture system.^[^ ^17^ ^]^ Interestingly, mutations impairing MyD88‐dependent TLR signaling decreased the number of IAHC cells but increased the number of HSCs in the AGM region of mouse embryos, indicating that the generation of IAHC and HSC are uncoupled, which might require complex physiological regulation.^[^ ^18^ ^]^ Several factors negatively regulating EHT have also been reported, such as the suppression of HEC specification by endothelial Sox17 and Smad4.^[^ ^19^, ^20^ ^]^ The absence of Ezh1, an epigenetic silencing regulator, also enhances hematopoietic multipotency in mouse embryos.^[^ ^21^ ^]^ Interestingly, other regulators, such as Dlk1 and p57Kip2, have also been reported to negatively regulate HSPC generation in a non‐cell‐autonomous manner.^[^ ^22^, ^23^ ^]^ Nevertheless, the molecular events and mechanisms restraining EHT are poorly understood.

Recently, the developmental trajectory and molecular signature of EHT have been elucidated using single‐cell transcriptomics.^[^ ^24^, ^25^, ^26^ ^]^ New intermediate cell populations and enriched markers have been identified.^[^ ^25^, ^26^, ^27^ ^]^ In our recent study, the HSC‐primed HEC population was identified using single‐cell transcriptomic analysis, and its developmental path from arterial endothelial cells (AECs) was further elucidated. These HSC‐primed HECs peak at E10.0 and can be efficiently captured by the surface marker combination PK44 (CD41^−^CD43^−^CD45^−^CD31^+^CD201^+^Kit^+^CD44^+^) based on single‐cell transcriptomic prediction and functional validation.^[^ ^25^, ^28^ ^]^ By comparison with other endothelial and CD45^−^ hematopoietic cell populations around the same developmental stages, a total of 11 signature genes of HSC‐primed HECs have been identified, including the well‐known HEC marker gene *Gfi1* and others such as *Neurl3* and *Nupr1*, the implication of which remains largely unknown. By further constructing a Neurl3‐EGFP reporter mouse model, we revealed that Neurl3 expression effectively enriched HSC‐primed HECs.^[^ ^25^ ^]^ These studies provide important clues for future research into the mechanisms underlying EHT and HSC formation. Notably, a recent study revealed that Nupr1, a stress‐responsive molecule involved in multiple biological contexts, is an adult HSC quiescence regulator that coordinates with the p53 signaling pathway.^[^ ^29^ ^]^ However, the physiological function of Nupr1 in blood generation during embryogenesis remains unknown.

Using a conditional gene knockout mouse model combined with single‐cell RNA‐seq (scRNA‐seq) and functional assays, we elucidated the negative role of endothelial Nupr1 in HEC specification and HSPC generation in the AGM region. Our findings uncovered a novel negative regulator of EHT and provide a new target for future HSC regeneration research.

## 2 Results

### 2.1 Loss of Endothelial Nupr1 Results in an Increase in HSPCs Generation in AGM Region

**Figure 1:**
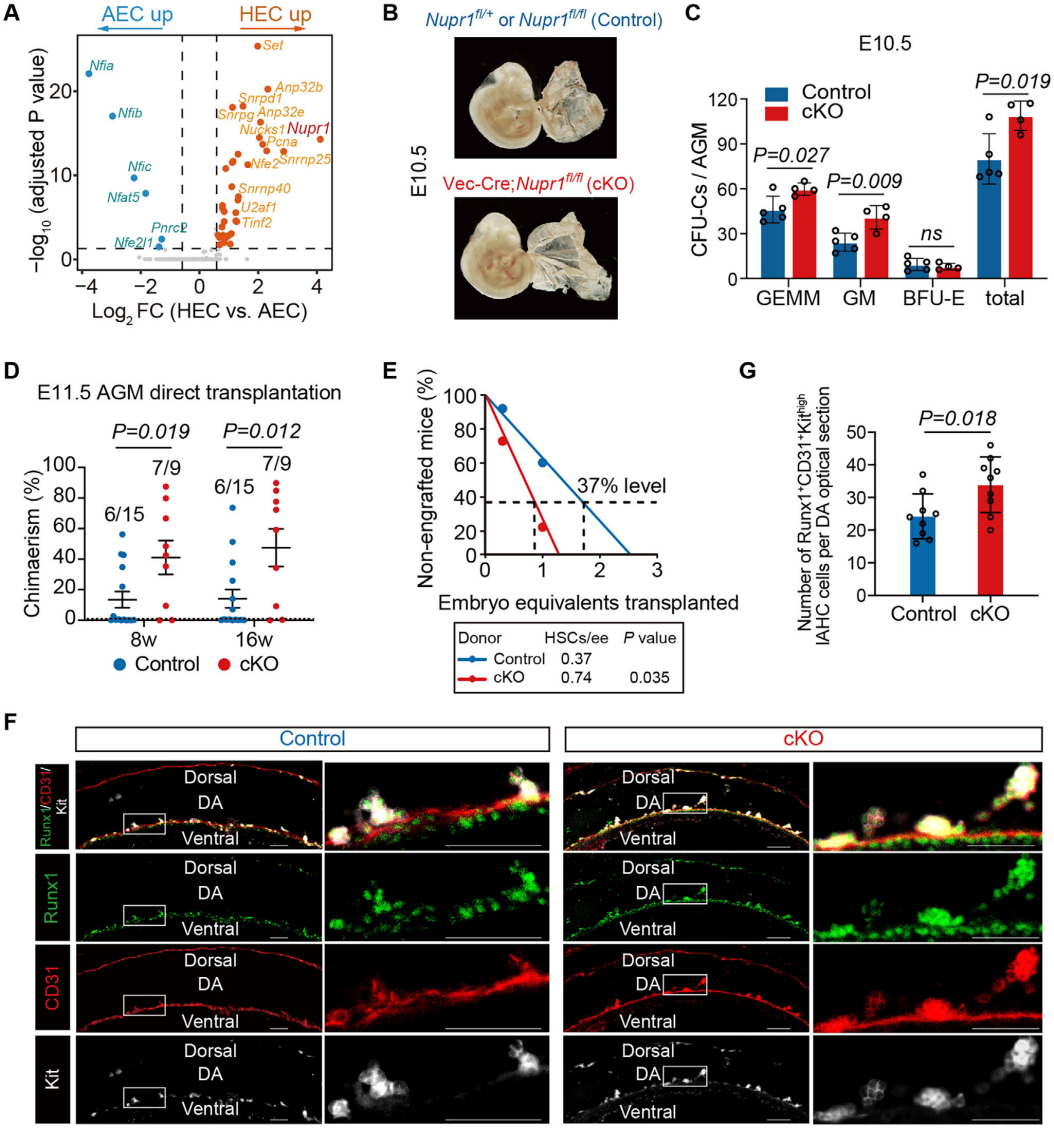
Loss of endothelial Nupr1 results in an increase in HSPCs generation in AGM region. A) Volcano plot showing differential expression level of genes encoding nuclear factors between AEC and HEC cells. B) Representative images of E10.5 (37‐39 sp) Control and cKO embryos. C) Number of CFU‐Cs in E10.5 AGM. Control (*n* = 5) and cKO (*n* = 4). Data are represented as mean ± SD and analyzed by unpaired two‐tailed Student's *t*‐test. D) Donor chimerism in peripheral blood of recipients after direct transplantation of E11.5 Control or cKO AGM cells (1 ee per recipient). Data are represented as mean ± SEM and analyzed by unpaired two‐tailed Student's *t*‐test. E) Quantification of HSCs in E11.5 Control and cKO AGM regions by limiting dilution assay. F) Representative whole‐mount confocal images of E9.5 (29‐30 sp) Control and cKO AGM regions stained with CD31, Runx1 and Kit (left). The boxed floor of the DA regions is shown at higher magnification (right). DA, dorsal aorta. Scale bars, 50 µm. G) Quantification of Runx1^+^CD31^+^Kit^high^ IAHC cells on each consecutive optical sections of DAs corresponding to (F). Three Control and three cKO embryos were analyzed. Data are represented as mean ± SD and analyzed by unpaired two‐tailed Student's *t*‐test.

As one of the signature genes of HSC‐primed HECs, *Nupr1* belongs to the nuclear factor genes. Therefore, we compared the expression of the genes encoding nuclear factors between the in silico‐identified and sorted HEC and AEC populations. We found that *Nupr1* had the largest fold change and was highly expressed in HECs compared with AECs (**Figure** **1**A and Figure S1A, Supporting Information). To further investigate the function of *Nupr1* in embryonic hematopoiesis, we generated Vec‐Cre;*Nupr1^fl/fl^* conditional knockout (cKO) embryos in which *Nupr1* was deleted from the endothelial cell stage. Littermate *Nupr1^fl/fl^* and *Nupr1^fl/+^* embryos were used as controls. No abnormal gross phenotype was observed in cKO embryos at E10.5–E11.0 (37–43 somite pairs) (Figure 1B). Notably, in the colony‐forming unit in culture (CFU‐C) assay, significantly increased total CFU‐C numbers were observed in the cKO AGM regions (Figure 1C), while no difference was detected in the yolk sac regions, which mainly due to rarely expression of *Nupr1* in yolk sac cells^[^ ^30^ ^]^ (Figure S1B,C, Supporting Information). Next, we performed transplantation experiments to evaluate the ability to generate adult repopulating HSCs in the AGM region with one embryo equivalent of E11.5 AGM cells transplanted into lethally irradiated adult recipient mice. The results showed significantly higher chimerism (47.50% ± 12.34%) in the peripheral blood at 16 weeks post‐transplantation when compared to the control group (14.15% ± 5.93%) with no difference in the lineage constitutions (Figure 1D and Figure S1D,E, Supporting Information). We next performed a limiting dilution assay, in which a cohort of recipient mice was transplanted with 0.3 embryo equivalent (ee) and 1.0 ee of dissociated E11.5 AGM cells from the control and cKO embryos. The results showed that the number of HSCs was increased nearly twofold in cKO embryos (at a frequency of 0.74/ee) compared to that in the controls (0.37/ee) (Figure 1E and Figure S1F, Supporting Information).

We then explored whether the enhanced hematopoietic activity in the AGM region was a result of the increased in situ generation of HSPCs in cKO embryos. We examined Runx1^+^CD31^+^Kit^high^ intra‐aortic hematopoietic cluster (IAHC) cells in the aortas of E9.5 embryos by whole‐mount staining. The results showed that cKO embryos had more IAHC cells than the controls (Figure 1F,G). This observation was further supported in E10.0 embryos, which consistently revealed that the number of Runx1^+^CD31^+^ cells, either in total or localized in IAHCs, were both remarkably increased in the cKO AGM region when compared to those in the controls (Figure S2A–C, Supporting Information). The finding was also verified by immunostaining on cross sections of the dorsal aorta at E9.5 and E10.0, showing a significantly increased number of Runx1^+^CD31^+^ cells attached to the multiple loci of endothelial layer in cKO sections when compared to controls (Figure S2D–F, Supporting Information). Taken together, these results reveal that *Nupr1* deletion in endothelial cells resulted in increased HSPC generation in the AGM region.

### 2.2 Loss of Endothelial Nupr1 Promotes HEC Specification in the AGM Region

**Figure 2:**
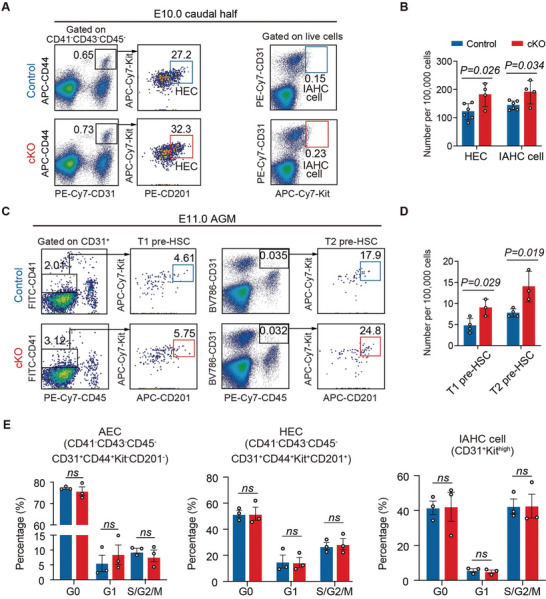
Loss of endothelial Nupr1 promotes HEC specification in the AGM region. A) Representative flow cytometric analysis of HEC (CD41^−^CD43^−^CD45^−^CD31^+^CD44^+^Kit^+^CD201^+^) and IAHC cells (CD31^+^Kit^high^) in E10.0 (32–34 sp) Control and cKO caudal half regions. B) Graph showing the number of HEC and IAHC cells in (A). Data are collected from four independent experiments. Data are represented as mean ± SD and analyzed by unpaired two‐tailed Student's *t*‐test. C) Representative flow cytometric analysis of T1 pre‐HSC (CD31^+^CD45^−^CD41^low^Kit^+^CD201^high^) and T2 pre‐HSC (CD31^+^CD45^+^Kit^+^CD201^high^) in E11.0 (41–43 sp) Control and cKO AGM regions. D) Graph showing the number of T1 and T2 pre‐HSCs in (C). Data are collected from three independent experiments. Data are represented as mean ± SD and analyzed by unpaired two‐tailed Student's *t*‐test. E) Histograms showing the percentage of G0, G1, or S/G2/M phases in AEC (CD41^−^CD43^−^CD45^−^CD31^+^CD44^+^Kit^−^CD201^−^), HEC (CD41^−^CD43^−^CD45^−^CD31^+^CD44^+^Kit^+^CD201^+^), and IAHC cells (CD31^+^Kit^high^) in E10.0 Control and cKO caudal half regions. Data are collected from three independent experiments. Data are represented as mean ± SEM and analyzed by unpaired two‐tailed Student's *t*‐test.

HSPCs originate from HECs through EHT through multiple steps including HEC specification, expansion, fate transition to HSPCs, HSPC proliferation, and differentiation. We then used flow cytometric analysis to explore the stages of EHT at which the defect occurred due to the absence of endothelial Nupr1. First, the number of HSC‐primed HECs represented by the PK44 population was significantly higher in cKO embryos than in control embryos at both E9.5 and E10.0 (**Figure** **2**A,B and Figures S3 and S4A,B, Supporting Information). Second, the number of immunophenotypic IAHC cells (CD31^+^Kit^high^) also increased in cKO embryos (Figure 2A,B and Figure S4A,B, Supporting Information), in line with the histological findings (Figure 1F,G and Figure S2, Supporting Information). Finally, the numbers of functionally T1 pre‐HSCs (CD31^+^CD45^−^CD41^low^Kit^+^CD201^high^) and T2 pre‐HSCs (CD31^+^CD45^+^Kit^+^CD201^high^) in the E11.0 AGM region were both significantly increased in cKO embryos compared to controls (Figure 2C,D and Figures S3 and S4G,H, Supporting Information). We also determined the numbers of HECs, IAHCs, and pre‐HSCs in haploinsufficient (Vec‐Cre;*Nupr1^fl/+^*) embryos at E10.0 and E11.0, and found no differences between control and haploinsufficient embryos (Figure S4C–F, Supporting Information).

Considering the potential role of Nupr1 as a negative regulator of the cell cycle and iron‐induced cell death (ferroptosis),^[^ ^29^, ^31^ ^]^ we first examined the cell cycle states of different EHT‐related populations at E10.0/E11.0, including immunophenotypic AECs, HECs and IAHC cells, by Hoechst and Ki67 staining. Notably, no difference in cell cycle status between control and cKO embryos was detected (Figure 2E and Figure S5A, Supporting Information). We also performed a BrdU incorporation assay, and found that the BrdU incorporation rates in these EHT‐related cell populations were unaffected (Figure S5B,C, Supporting Information), further confirming that Nupr1 had little influence on the cell cycle status. We then examined the cell death status of the EHT‐related cells (CD31^+^CD45^−^Kit^+^) using Annexin V/7‐AAD staining, which is frequently used to identify both cell apoptosis (Annexin V^+^) and ferroptosis (7‐AAD^+^).^[^ ^32^ ^]^ The results indicated that there was no significant difference in cell death status between control and cKO embryo (Figure S5D,E, Supporting Information). Collectively, these results suggest that loss of endothelial Nupr1 promotes HEC specification, leading to a consequently increased generation of HSPCs, rather than affects their proliferation ability in the AGM region.

### 2.3 scRNA‐Seq Reveals a Promoted Transition from AECs to EHT Cells in Nupr1‐Deficient Embryos

**Figure 3:**
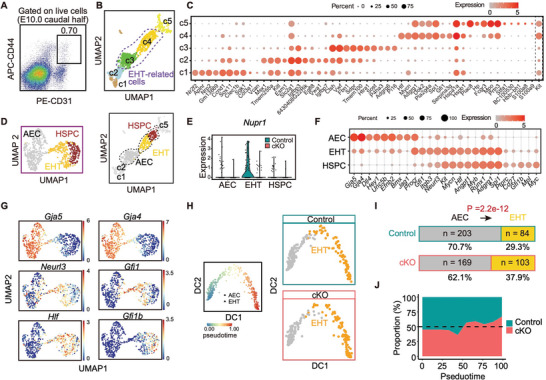
scRNA‐seq reveals a promoted transition from AECs to EHT cells in Nupr1‐deficient embryos. A) Representative flow cytometric analysis of CD31^+^CD44^+^ cells in E10.0 (32–33 sp) Control and cKO AGM regions. B) UMAP plot showing the cells of unsupervised clustering of integrated data. The clusters (c3 and c4) outlined by dashed lines were used for subsequent analysis. C) Dot plot displaying the expression levels of top 10 signature genes in five clusters in (B) and Kit as a reference. D) UMAP plot showing three subclusters were distinguished from c3 and c4 (left). UMAP plot showing the three subclusters map into c1–c5 clusters (right). E) Violin plot showing the expression levels of *Nupr1* in AEC, EHT, and HSPC clusters. F) Dot plot displaying the expression levels of signature genes in in AEC, EHT, and HSPC clusters. G) UMAP plot showing the expression level of indicated genes in in AEC, EHT, and HSPC clusters. H) Trajectory analysis indicating the developmental path from AEC to EHT cells, and the EHT cells along the pseudotime were shown. I) Bar chart revealing the distribution of cells in AEC, EHT clusters in Control and cKO embryos. The chi‐square test was used to evaluate the constitution differences between Control and cKO cells. J) Aera plot showing the proportion along the development pseudotime from AECs to EHT cells between control and cKO embryos.

There could be potential differences between immunophenotype and identification at the whole transcriptomic level of given cell populations, especially when the representativeness of immunophenotypes might be compromised upon the genetic ablation of a functional gene. Considering this, we performed scRNA‐seq of CD31^+^CD44^+^ cells from the E10.0 caudal half region (**Figure** **3**A and Figure S6B,C, Supporting Information), which possibly contains AECs of the dorsal aorta and the predominant populations involved in EHT.^[^ ^25^, ^33^ ^]^ Cells from littermate control and cKO embryos were labeled with different hashtag oligos, sorted, and pooled together for 10× genomics scRNA‐seq with two independent replicates (Figure S6A,B, Supporting Information). After quality control and batch effect removal, 2139 cells were retained, which were robustly divided into three major cell populations (C1–C3) by unsupervised clustering (Figure S6D–F, Supporting Information). *Nupr1* deletion did not result in either the generation or in the loss of any cell population (Figure S6D, Supporting Information). C1 showed a high expression of the endothelial marker *Cdh5* and arterial gene *Gja5* thus, we focused on subsequent exploration. C2 and C3 were recognized as megakaryocytes and macrophages, respectively, given their high expression of the corresponding markers and their enriched biologic functions (Figure S6F,G, Supporting Information),^[^ ^34^, ^35^, ^36^, ^37^ ^]^ both of which were excluded from further analysis.

C1 was further subdivided into five cell clusters, c1–c5 (Figure 3B). Of these, two small endothelial populations, c1 and c2, were characterized by *Kitl* expression (Figure 3C), which was previously identified to be localized at the vascular plexus but not major arteries.^[^ ^25^ ^]^ c5 highly expressed genes enriched in myeloid progenitors (*Mpo* and *Fcgr3*) (Figure 3C). These three clusters were not directly relevant to the EHT and were therefore removed. In contrast, c3 was characterized by the high expression of genes expressed majorly in the arteries (*Tmem100*, *Htra1*, and *Adgrg6*),^[^ ^38^ ^]^ and c4 highly expressed several signature genes of HSC‐primed HECs and pre‐HSCs (*Hlf*, *Mycn*, *Adgrg1*, and *Gfi*) (Figure 3C).^[^ ^25^, ^39^ ^]^ Thus, c3 and c4 were recognized as EHT‐related cells and were selected for further analysis, which included 363 and 332 individual cells from the control and cKO embryos, respectively.

Re‐clustering of EHT‐related cells showed that three subclusters could be distinguished (Figure 3D); c3 corresponded to the AEC population, and c4 was further divided into two subclusters: EHT and HSPC (Figure 3D). The EHT cluster retained a weak expression of arterial genes, a high expression of the HEC markers *Gfi1* and *Neurl3*, and importantly, specifically the expression of *Nupr1* (Figure 3E–G). Therefore, the EHT cluster represented HECs and the cells undergoing EHT, which correspond to the immunophenotypic PK44 cells, given their exclusive co‐expression of *Procr* and *Kit* among the three EHT‐related cell populations (Figure 3F). We further confirmed the sharply decreased expression of *Nupr1* in cells from cKO embryos compared to that in controls (Figure 3E). Pseudotime trajectory analysis demonstrated a continuous development path from AEC to EHT cluster cells, representing the critical fate transformation seen in EHT (Figure 3H). Importantly, a promoted transition from AECs to EHT cells was observed in Nupr1‐deficient embryos, showing a relatively decreased proportion of AECs and an increased proportion of EHT cells compared to the controls (Figure 3H–J). Therefore, the scRNA‐seq data validated the findings of flow cytometric and histological analyses, showing an enhanced HEC specification in Nupr1‐deficient embryos. Considering that Nupr1 is also expressed in a small portion of AECs, these results suggest that Nupr1 has begun to function at this early stage of HEC specification (Figure 3E).^[^ ^25^ ^]^

### 2.4 Loss of Nupr1 Facilitates Blood‐Forming Capacity of HECs

**Figure 4:**
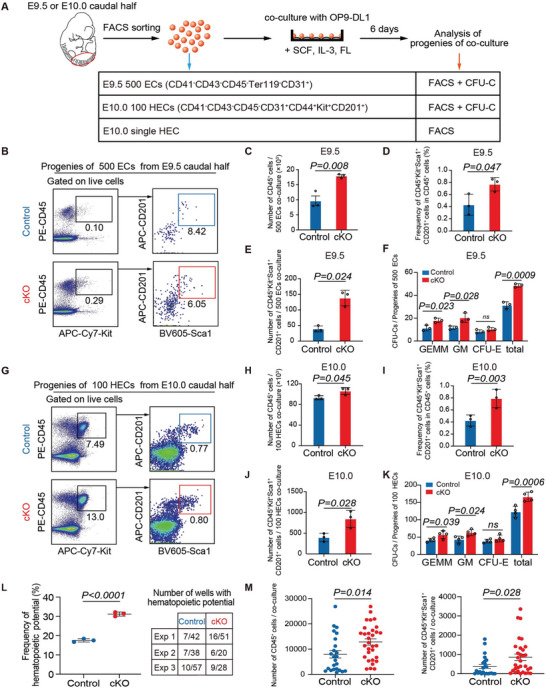
Loss of Nupr1 facilitates blood‐forming capacity of HECs. A) Schematic illustration of hematopoietic induction of ECs or HECs from E9.5 (26–28 sp) or E10 (32–34 sp) embryos. B) Representative flow cytometric analysis of the progenies of E9.5 ECs co‐culture. C) Quantification of the number of hematopoietic cells (CD45^+^) generated from E9.5 ECs co‐culture. Data are collected from three independent experiments. Data are represented as mean ± SD and analyzed by unpaired two‐tailed Student's *t*‐test. D,E) Quantification of the frequency and the number of CD45^+^Kit^+^Sca1^+^CD201^+^ cells generated from E9.5 ECs co‐culture. Data are collected from three independent experiments. Data are represented as mean ± SD and analyzed by unpaired two‐tailed Student's *t*‐test. F) CFU‐C analysis of the derivatives of E9.5 ECs co‐culture. Data are collected from three independent experiments. Data are represented as mean ± SD and analyzed by unpaired two‐tailed Student's *t*‐test. G) Representative flow cytometric analysis of the progenies of E10.0 HECs co‐culture. H) Quantification of the number of hematopoietic cells (CD45^+^) generated from E10.0 HECs co‐culture. Data are collected from three independent experiments. Data are represented as mean ± SD and analyzed by unpaired two‐tailed Student's *t*‐test. I,J) Quantification of the frequency and the number of CD45^+^Kit^+^Sca1^+^CD201^+^ cells generated from E10.0 HECs co‐culture. Data are collected from three independent experiments. Data are represented as mean ± SD and analyzed by unpaired two‐tailed Student's *t*‐test. K) CFU‐C analysis of the derivatives of E10.0 HECs co‐culture. Data are collected from three independent experiments. Data are represented as mean ± SD and analyzed by unpaired two‐tailed Student's *t*‐test. L) Graph showing the frequencies of wells with hematopoietic potential after E10.0 single HEC co‐culture. Data are collected from three independent experiments. Data are represented as mean ± SD and analyzed by unpaired two‐tailed Student's *t*‐test. Number of wells with hematopoietic potential in individual experiment was shown in the right table. M) Quantification of the number of CD45^+^ cells (left) and CD45^+^Kit^+^Sca1^+^CD201^+^ cells (right) in each well with hematopoietic potential after E10.0 single HEC co‐culture. Data are represented as mean ± SD and analyzed by unpaired two‐tailed Student's *t*‐test.

Next, we performed a series of functional assays to determine the hemogenic capacity of HECs in Nupr1 deficiency. Briefly, candidate endothelial populations were isolated and co‐cultured with OP9‐DL1 stromal cells for six days in a culture system similar to that previously used for inducing HSC‐primed HECs or pre‐HSCs to mature into transplantable HSCs (**Figure** **4**A).^[^ ^25^, ^39^ ^]^ First, we evaluated the blood‐forming capacity of the whole endothelial pool from the caudal half in the E9.5 embryo. By performing co‐culture using 500 immunophenotypic endothelial cells, we detected more CD45^+^ hematopoietic cells in cKO embryos than in control embryos (Figure 4B,C). Of note, we found that the generation of immunophenotypic HSPCs (CD45^+^Sca1^+^Kit^+^CD201^+^) was dramatically increased in cKO cultures (Figure 4D,E). This finding was further confirmed by the CFU‐C assay, which showed an increase in hematopoietic progenitors generated from the endothelial cells of E9.5 cKO embryos (Figure 4F). Considering that, compared with controls, endothelial cells from cKO embryos contained more immunophenotypic HECs (PK44 cells) (Figure 2A) and more EHT population as detected by scRNA‐seq (Figure 3H,I), the increased hematopoietic products might be at least in part due to the increased HEC proportion in the endothelial pool. Next, to determine whether the hemogenic capacity of HECs was altered by Nupr1 deficiency, we co‐cultured 100 immunophenotypic HECs (PK44 cells) from the caudal half in E10.0 embryos. Notably, more CD45^+^ hematopoietic cells were generated (Figure 4G,H) along with immunophenotypic HSPCs and hematopoietic progenitors, as detected by the CFU‐C assay (Figure 4I–K). Therefore, the blood‐forming capacity of HECs was enhanced in the absence of Nupr1.

In our previous studies, HECs represented by PK44 population have been transcriptomically and functionally proven to have initiated their intrinsic hemogenic program featured by Runx1 expression and present a continuum of cellular states from endothelial‐biased characteristics to hematopoietic‐biased characteristics prior to acquiring hematopoietic function.^[^ ^25^, ^28^, ^40^ ^]^ To determine whether the proportion of cells with blood‐forming capacity was increased in PK44‐represented HECs, or if the hemogenic capacity of individual blood‐forming cells was enhanced, or both, we performed single HEC induction and functional assays. First, 31.3% (31/99) of cKO PK44 cells gave rise to hematopoietic progenies, unlike only the 17.5% (24/137) of the controls, indicating a 1.8‐fold increase (Figure 4L). Second, the numbers of CD45^+^ hematopoietic products and immunophenotypic HSPCs generated per blood‐forming cell were both increased in the HECs from cKO embryos compared to controls (Figure 4M). Taken together, these functional data revealed that loss of endothelial Nupr1 promoted the specification of hematopoiesis‐primed functional HECs as well as enhanced the hemogenic potential of individual HECs toward HSPCs, consisting with the findings revealed by our single‐cell transcriptome data (Figure 3J).

### 2.5 Elevated TNF‐*α* Contributes to the Increased HSPC Generation in Nupr1‐Deficient Embryos

**Figure 5:**
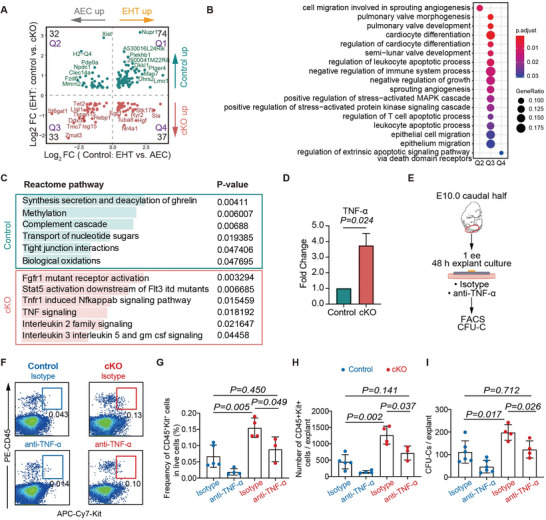
Elevated TNF‐*α* contributes to the increased HSPC generation in Nupr1‐deficient embryos. A) Dot plot displaying the differentially expressed genes (*x*‐axis) between AEC (left) or EHT (right) and differentially expressed genes upon loss of Nupr1 in EHT cells (*y*‐axis). Number of genes in each quadrant was labeled in the corner. The genes with *P‐*value less than 0.05 and fold change bigger than 1.3 were selected as differentially expressed genes and were labeled in dark green (up regulated genes in Control cells) or red (upregulated genes in cKO cells). B) Dot plot showing the enriched GO terms for Q1, Q2, Q3, and Q4 in (A), respectively (the genes in Q1 were not enriched in any GO term). C) Enriched Reactome pathways were shown between Control and cKO EHT cells. Wilcox test is used to assess the difference between the two groups, and *P*‐value less than 0.05 is considered statistically significant. D) qRT‐PCR detecting the expression level of TNF‐*α* in sorted EHT cells (CD31^+^CD45^−^Kit^+^) from E10.0 Control and cKO caudal half regions. Data are collected from two independent experiments. E) Schematic of explant cultures of E10.0 Control and cKO caudal half regions with isotype or anti‐TNF‐*α*. F) Representative flow cytometric analysis of the hematopoietic productions after explant cultures. G,H) Quantification of the frequency and the number of CD45^+^Kit^+^ cells from the derivatives of explant cultures. Data are collected from three independent experiments. Data are represented as mean ± SD and analyzed by unpaired two‐tailed Student's *t*‐test. I) CFU‐C analysis of the derivatives of explant cultures of E10.0 Control and cKO caudal half regions. Data are collected from three independent experiments. Data are represented as mean ± SD and analyzed by unpaired two‐tailed Student's *t*‐test.

Taking advantage of the scRNA‐seq data, we explored the potential mechanism underlying the promoted EHT by Nupr1 deficiency. First, we evaluated the extent to which Nupr1 deficiency affects normal EHT‐related molecular changes. We found that a set of differentially expressed genes (DEGs) between cKO and control cells in the EHT population were also a part of the DEGs between AEC and EHT clusters in control embryos (**Figure** **5**A). Notably, several hematopoietic transcription factors, such as *Egr1* and *Nr4a1*, were expressed more in cKO EHT cells (Figure 5A). Among these, Nr4a1 has been reported to maintain the function of HSCs and lymphocytes. Gene Ontology terms were mainly enriched in the genes upregulated in cKO compared with control EHT cells, and simultaneously belonged to the genes that were expected to be downregulated during the EHT process (Figure 5B). This finding suggests that the enhanced hemogenic activity resulting from Nupr1 deficiency was accompanied by the retention of certain characteristics of the upstream arterial endothelial populations.

Remarkably, Reactome signaling pathway analysis revealed that several inflammatory signals were overrepresented in Nupr1‐deficient EHT cells compared with controls, including TNF signaling, TNFR1 induced NF‐*κ*B signaling pathway, and their downstream signals, such as Interleukin 2, Interleukin 3, and Interleukin 5 signaling (Figure 5C). We mapped the overall expression levels of the genes associated with these signaling pathways onto the pseudotime axis of the EHT process and found that they were maintained or slightly elevated during EHT in the controls, whereas they were increased by Nupr1 deficiency in the EHT population (Figure S7A,B, Supporting Information). Previous studies have revealed that elevated levels of these pro‐inflammatory cytokines dramatically promote HEC specification and HSPC generation. We then analyzed the expression levels of several pro‐inflammatory cytokines in our single‐cell transcriptome data (Figure S7C, Supporting Information). Notably, we found that one of the most important proinflammatory cytokines, tumor necrosis factor‐*α* (TNF‐*α*), was significantly upregulated in Nupr1‐deficient EHT cells, which was further confirmed by qRT‐PCR validation in sorted control and cKO E10.0 AGM EHT‐ related cells (CD31^+^CD45^−^Kit^+^) (Figure 5D). To further validate whether the elevated TNF‐*α* may contribute to the increased HSPC generation in Nupr1‐deficient embryos, we added a specific TNF‐*α* neutralizing antibody to block its function in an explant culture assay (Figure 5E). Notably, the results showed that the proportion and number of HSPCs generated was reduced back to the levels in control embryos after TNF‐*α* neutralization in cKO embryos (Figure 5F–I).

Taken together, these data suggested that Nupr1 restrained the EHT process mainly by participating in the modification of TNF‐*α* mediated inflammatory signals. However, the precise underlying regulatory mechanisms need to be explored in the future.

## 3 Discussion

Uncovering the precise molecular events and regulatory mechanisms underlying EHT and HSC development will provide important resources and novel targets for in vitro regeneration of transplantable HSCs. Current studies have mainly focused on the positive regulators of EHT, whereas relatively few suppressors have been discovered.

In our study, we found that specific deletion of endothelial Nupr1 leads to increased generation of HECs and HSPCs in the AGM region. Subsequently, in vivo and in vitro experiments confirmed that the loss of endothelial Nupr1 generates more functional HECs and HSPCs. Recently it was found that Nupr1 is highly expressed in adult HSCs, after the deletion of which, their function was significantly enhanced, showing augmented long‐term remodeling ability and multilineage differentiation potential. Mechanistically, Nupr1 regulates the proliferation of quiescent HSC by inhibiting the p53 signaling pathway.^[^ ^29^ ^]^ Nupr1 has also been reported to be involved in cell death, mitochondrial dysfunction, and ROS production under various stress conditions.^[^ ^41^ ^]^ Notably, in the present study, we found that several TNF‐related inflammatory signaling pathways were significantly up‐regulated in Nupr1‐deficient cells through scRNA‐seq analysis, which has not been previously reported, suggesting the existence of distinct regulatory mechanisms that might be cell‐context‐dependent. Through functional validation, we found that TNF‐*α* was upregulated in Nupr1‐deficient HECs and that the use of a neutralizing antibody could partially restore the excessive HSPC production in the explant cultures from Nupr1‐deficient embryos. However, whether Nupr1 directly regulates TNF‐*α* expression, and the underlying mechanism remain unclear. Further studies are required to explore these molecular details.

In summary, our findings reveal that Nupr1 is an important negative regulator of EHT. Combined with its function in adult HSCs, we believe that Nupr1 may be an ideal target for HSC regeneration research in future.

## 4 Experimental Section

### Mice

*VEC‐Cre* mice were purchased from Jackson Laboratory (B6;129‐Tg(Cdh5‐cre)1Spe/J). *Nupr1^fl/fl^* mice were described previously.^[^ ^29^ ^]^ All mice were maintained on C57BL/6 genetic background and bred in Specific Pathogen Free (SPF) condition at the Laboratory Animal Center of Academy of Military Medical Sciences. E9.5–E11.5 embryos were confirmed by counting the somite pairs (sp). The caudal half or AGM region was dissected as previously reported.^[^ ^7^ ^]^ The experimental manipulations of mice were approved by the Animal Care and Use Committee of the Institute.

### OP9‐DL1 Co‐Culture and Colony Forming Unit‐Culture (CFU‐C) Assay

FACS‐sorted cells were co‐cultured with OP9‐DL1 stromal cells in 24‐well plate, containing *α*‐MEM (Gibco), 10% fetal bovine serum (Hyclone), and cytokines (100 ng mL^−1^ SCF, 100 ng mL^−1^ Flt3 ligand, and 100 ng mL^−1^ IL‐3, PeproTech). After 6 d of co‐culture, cells in each well were harvested for flow cytometry analysis. For CFU‐C assay, cells were plated in 35 mm Petri dish containing 1.5 mL methylcellulose‐based medium with recombinant cytokines (MethoCult GF M3434, STEMCELL Technologies) at 37 °C, 5% CO_2_ in a humidified chamber. Colonies were quantified after 7 d.

### Immunofluorescence and Whole‐Mount Immunostaining

The immunofluorescence and whole‐mount immunostaining assays were performed as previously reported.^[^ ^25^ ^]^ Briefly, for immunofluorescence, embryos were isolated, fixed with 4% paraformaldehyde, and embedded in paraffin in the sitting position (rostrum up, caudal part down). Then, using a Leica RM2235, the trunk region of E9.5/E10.0 embryos was consecutively sectioned at a range of 5–6 µm between the forelimb buds and the hindlimb buds. Considering the intra‐aortic clusters were particularly abundant in middle region of the dorsal aorta,^[^ ^42^ ^]^ the median slices among all the consecutive slices were picked for subsequent counting. For instance, after sectioning the trunk between the forelimb and hindlimb buds, a total of 30 slices were acquired, of which slices 11–20 were taken into account for further counting. For each E9.5/E10.0 control embryo, eight sections were counted, and for each E9.5/E10.0 cKO embryo, ten sections were counted. For whole‐mount immunostaining, embryos were isolated and fixed with 2% paraformaldehyde, then the body parts between forelimb buds and hindlimb buds of E10.0 embryos were stained by antibodies in accordance with the standard protocol.

### Processing of scRNA‐Seq Data

Sequencing data from 10X genomics was processed with CellRanger software (version 5.0.1) with default mapping arguments. First, quality control was performed to filter out low quality cells. Then, the cells with singlet hashtag labels were selected using HTODemux function in Seurat^[^ ^43^ ^]^ (Version 4.1.0) package with default parameters. The cells were retained as following criteria: 1) more than 1800 genes, 2) more than 6000 UMIs, and 3) less than 5% of reads mapped to mitochondrial genes. Seurat (version 4.1.0) was used for further analyzes. Briefly, FindVariableGenes function was used to select highly variable genes (HVGs) with default parameters. HVGs were used as input for PCA dimension reduction. The top relevant PCs selected by Elbow methods were used for UMAP and unsupervised graph‐based clustering.

### Identification of DEGs and GO Enrichment

Genes detected in a minimum fraction of 0.1 in either of two cell populations were used for identify DEGs. FindMarkers function was employed to identify DEGs with Wilcoxon rank sum test. Genes with fold‐change ≥ 1.3 and *P*‐value ≤ 0.05 were selected as DEGs. ClusterProfiler^[^ ^44^ ^]^ (version 4.0.5) was used to perform gene ontology biological process enrichment analysis. FDR was used to adjust the Hypergeometric test *P*‐value, and adjusted *P*‐value less than 0.05 was selected as cluster specific enriched GO term.

### Statistical Analysis

No statistical methods were used to predetermine sample size. For statistical analysis between groups, unpaired two‐tailed Student's *t*‐test was used to calculate *P‐*values, unless otherwise specified. Statistical analysis was carried out using GraphPad Prism 8.

## Conflict of Interest

The authors declare no conflict of interest.

## Author Contributions

H.W., D.L., and H.C. contributed equally to this work. Y.L., B.L., and J.Z. conceived and designed the study; H.W. and H.C. performed cell sorting with the help from Y.N.; H.W. performed co‐culture and transplantation assay with the help from S.H. and H.Z.; H.C. performed immunostaining assay with the help from Y.J. and R.Z.; H.W. and J.Z. performed scRNA‐seq; D.L. performed bioinformatics analysis with the help from Z.L.; J.W. kindly provided *Nupr1^fl/fl^* mice; Y.L. and J.Z. wrote the manuscript. All authors reviewed the manuscript.

## Supporting information

Supporting Information

## Data Availability

The scRNA‐seq data can be obtained in NCBI's Gene Expression Omnibus (GEO) with the accession number GSE205595. The data that support the findings of this study are available from the corresponding author upon reasonable request.
