# Supplementary material for: Nupr1 Negatively Regulates Endothelial to Hematopoietic Transition in the Aorta‐Gonad‐Mesonephros Region
Source: Adv Sci (Weinh). 2023 Jan 13;10(6):2203813. doi: 10.1002/advs.202203813 (PMC9951349; doi:10.1002/advs.202203813)
Supplement: Supplementary file 1 — Supporting Information [file ADVS-10-2203813-s001.pdf]

ADVANCED SCIENCE
Open Access

Supporting Information

for *Adv. Sci.*, DOI 10.1002/advs.202203813

Nupr1 Negatively Regulates Endothelial to Hematopoietic Transition in the Aorta-Gonad-Mesonephros Region

Haizhen Wang, Di Liu, Haifeng Chen, Yuqing Jiao, Haixin Zhao, Zongcheng Li, Siyuan Hou, Yanli Ni, Rong Zhang, Jinyong Wang, Jie Zhou, Bing Liu* and Yu Lan**

Supplementary methods

Flow Cytometry

Cells were sorted and analyzed by flow cytometers FACS Aria 2 and Calibur (BD Biosciences), and the data were analyzed with FlowJo software (Tree Star). Following antibodies were used for cell staining: B220 (eBioscience, RA3-6B2), CD3 (eBioscience, 145-2C11), CD4 (eBioscience, GK1.5), CD8 (eBioscience, 53-6.7), Ly-6G (BioLegend, 1A8), Mac1 (eBioscience, M1/70), CD31 (BD or BioLegend, MEC13.3), CD44 (eBioscience or BioLegend, IM7), CD41 (BD or eBioscience, MWReg30), CD43 (BD, S7), CD45 (eBioscience, 30-F11), CD45.1 (eBioscience, A20), CD45.2 (eBioscience, 104), CD201 (eBioscience, eBio1560), Kit (eBioscience, 2B8 or BioLegend, ACK2), Sca-1 (eBioscience, D7), Ter119 (eBioscience, TER-119), and 7-amino- actinomycin D (7-AAD; eBioscience), TotalSeq™-B0953 PE Streptavidin (Biolegend, Cat#405289), TotalSeq™-B0954 PE Streptavidin (Biolegend, Cat#405291).

Cell cycle analysis

For cell cycle analysis by Hoechst/Ki67 staining, cells were fixed using Fixation and Permeabilization Solution (BD, 554722). After surface marker antibodies staining, anti-Ki67-FITC (eBioscience, 20Raj1) and Hoechst 33342 (BD, 561908) staining were performed following standard protocol. For BrdU incorporation assay, 2 mg BrdU solution was injected intra-peritoneally into

pregnant females. Two hours later, embryos were collected and analyzed by APC BrdU Flow Kit (BD Pharmingen, 557892), following manufacturer's instructions.

AGM cells transplantation assay

Ten to twelve-week-old female recipients (CD45.1/1 mice) were subjected to a split dose of 9 Gy γ-irradiation (^{60}Co) at an interval of two hours. Freshly dissected E11.5 AGM cells (1ee or 0.3ee per recipient), together with 2×10^4 nucleated bone marrow cells (CD45.1/2), were injected into irradiated recipients (CD45.1/1) via the tail vein. Donor (CD45.2/2) chimerism in peripheral blood from recipients were detected at 8 and 16 weeks post transplantation. The recipients demonstrating ≥1% donor-derived chimerism in peripheral blood were counted as successfully reconstituted. HSC frequencies were determined by extreme limiting dilution analysis (ELDA)[1]. For transplantation of co-cultured cells from T1 and T2 pre-HSCs, the recipients demonstrating ≥5% donor-derived chimerism in peripheral blood were counted as successfully reconstituted.

Explant cultures

Caudal half regions were isolated from E10.0 embryos and cultured using an ex vivo explant culture system as previously described[2]. The medium used for the caudal half region culture contained IMDM (Hyclone) and 20% fetal bovine

serum (Hyclone) in the presence or absence of 10 μg/mL of TNF-α neutralizing antibody (anti-TNF-α) (eBioscience, TN3-19.12). After being cultured for 48 hours, caudal half regions were dissociated in collagenase for flow cytometry analysis and CFU-C assay.

FACS and cell hashing for scRNA-seq

Cells from each E10.0 control and cKO caudal half were resuspended in 50 μL FACS buffer (1×PBS with 1% BSA). Sorting antibodies cocktail was prepared and added to the cell suspension, and incubated for 30 min at 4 °C. After washing, each oligo barcoded streptavidin with titrated dosage (0.125 μL for 1 million cells) were incubated with the stained cells for 20 min at 4 °C. After washing 3 times, 10 nmol/L Biotin was added to bind redundant streptavidin in the cell suspension, and incubated for about 5min at 4 °C. After washing 3 times in 0.1% BSA/PBS, staining with 7-AAD was performed for 5 min. Cells from each embryo were sorted and pooled into one 1.5 mL centrifuge tube. Cells were then centrifuged at 400× *g* at 4 °C for 5 min, resuspend in 50 μL 0.04% BSA/PBS to load into one lane of a 10× Chromium V3 chip. The cDNA preparation was performed following the instruction manual, and the hashtag library was prepared following the BioLegend TotalSeq-B guide. The final cDNA library and tag library were both sequenced on NovaSeq 6000. Cells were sequenced to an average depth of 0.1 M per cell for cDNA and 0.01 M per cell for hashtags. For qRT-PCR, pools of 100 cells were sorted, reverse transcription

and cDNA amplification were performed as previously reported[3]. qRT–PCR was performed on LightCycler® 480 II system (Roche Diagnostics). The sequences of primers were: Nupr1: CCCTTCCCAGCAACCTCTAA (sense), AGCTTCTCTCTTGGTCCGAC (anti-sense); TNF-α: GATCTCAAAGACAACCAACATGTG (sense), CTCCAGCTGGAAGACTCCTCCCAG (anti-sense).

Integrated analysis of two experiments data

To eliminate batch effect, we employed the method of integration provided in Seurat for scRNA-seq. First, the features that repeatedly variable across two experiment datasets were selected for integration. Then, anchors were identified among variable features cross two datasets used for correcting batch effect.

Constructing single-cell trajectories

Diffusion pseudotime[4] (DPT) analyses in Scanpy[5] (version 1.8.1) was used to presume developmental trace. Briefly, a transition matrix by convolving Gaussians centered at nearby cells was built to construct the weighted nearest neighbor graph of the data. The probabilities of transition to each cell in the data was determined using random walks of any length on the nearest neighbor graph. Finally, the DPT between two cells was calculated with the Euclidian distance based on the probabilities vector.

Calculation of pathway activity

We employed AUCell[6] package to evaluate pathway activity of each cell for all Reactome pathways. Wilcox test was used to select population specific pathways.

Reference

[1] Hu Y, Smyth GK, *J Immunol Methods*. **2009**, 347, 1-2.

[2] Medvinsky A, Dzierzak E, *Cell*. **1996**, 86, 6.

[3] Gao S, Yan L, Wang R, Li J, Yong J, Zhou X, Wei Y, Wu X, Wang X, Fan X, Yan J, Zhi X, Gao Y, Guo H, Jin X, Wang W, Mao Y, Wang F, Wen L, Fu W, Ge H, Qiao J, Tang F, *Nat Cell Biol*. **2018**, 20, 10.

[4] Haghverdi L, Buttner M, Wolf FA, Buettner F, Theis FJ, *Nat Methods*. **2016**, 13, 10.

[5] Wolf FA, Angerer P, Theis FJ, *Genome Biol*. **2018**, 19, 1.

[6] Aibar S, Gonzalez-Blas CB, Moerman T, Huynh-Thu VA, Imrichova H, Hulselmans G, Rambow F, Marine JC, Geurts P, Aerts J, van den Oord J, Atak ZK, Wouters J, Aerts S, *Nat Methods*. **2017**, 14, 11.

Supplementary Figures

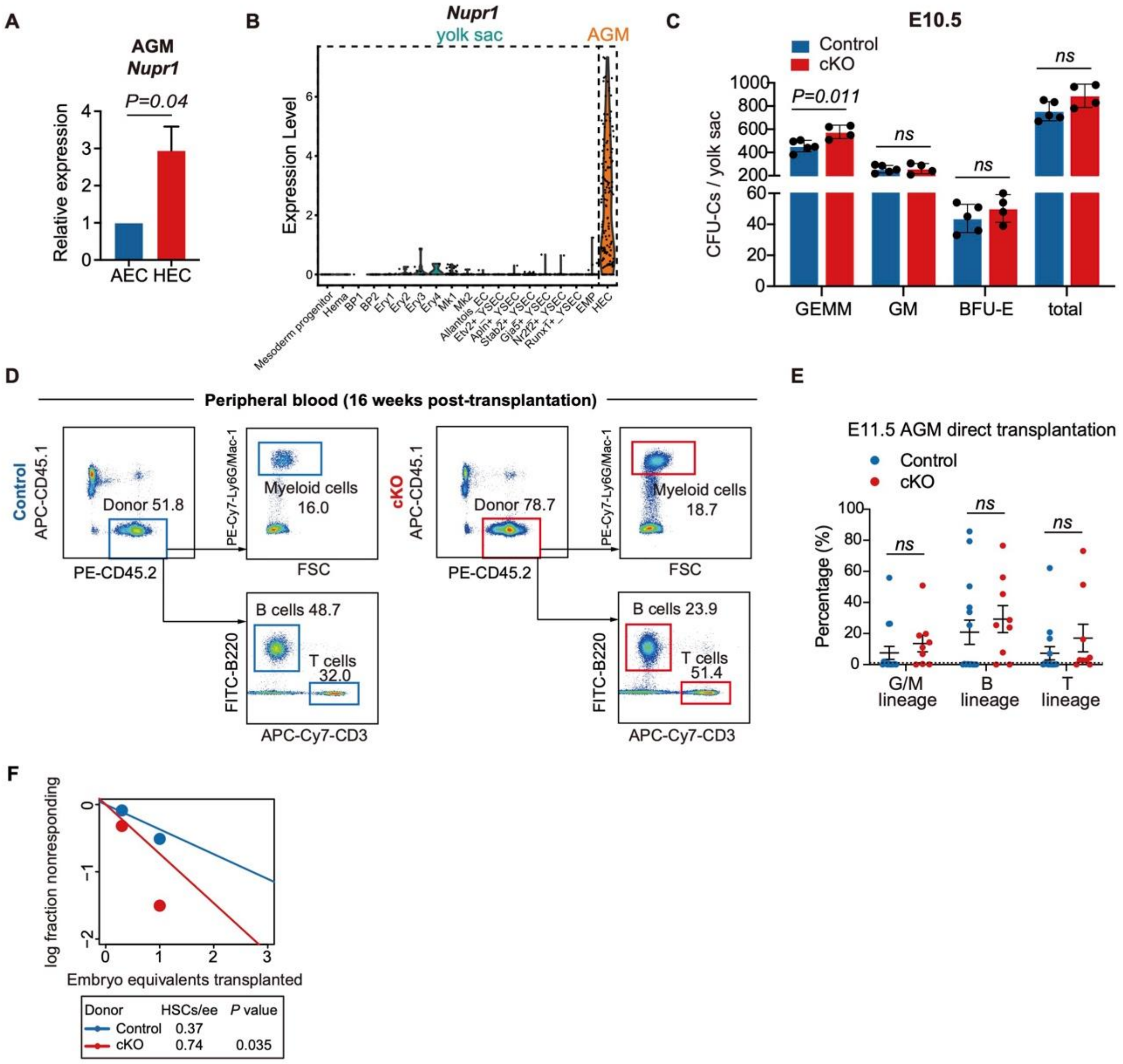

Supplementary Figure 1. Loss of Nupr1 led to an increase in HSPC generation in AGM region.

(A) The expression level of *Nupr1* in sorted AECs and HECs from E10.0 wild-type caudal half regions. Data are collected from 2 independent experiments.

(B) The expression level of *Nupr1* in distinct cell populations from yolk sac and HEC from AGM.

(C) Number of CFU-Cs in E10.5 yolk sac. Control (n=5) and cKO (n=4). Data

are represented as mean ± SD and analyzed by unpaired two-tailed Student's t test.

(D) Representative flow cytometric showing donor-derived ($CD45.2^+$) myeloid ($Ly6G^+$/$Mac\text{-}1^+$), B lymphoid ($B220^+$), and T lymphoid ($CD3^+$) cells in peripheral blood.

(E) Donor chimerism of myeloid ($Ly6G^+$/$Mac\text{-}1^+$), B lymphoid ($B220^+$), and T lymphoid ($CD3^+$) cells in peripheral blood after 16 weeks of transplantation. Data are represented as mean ±SEM.

(F) Quantification of HSCs in E11.5 Control and cKO AGM regions by limiting dilution assay with Y axis being log10 scale.

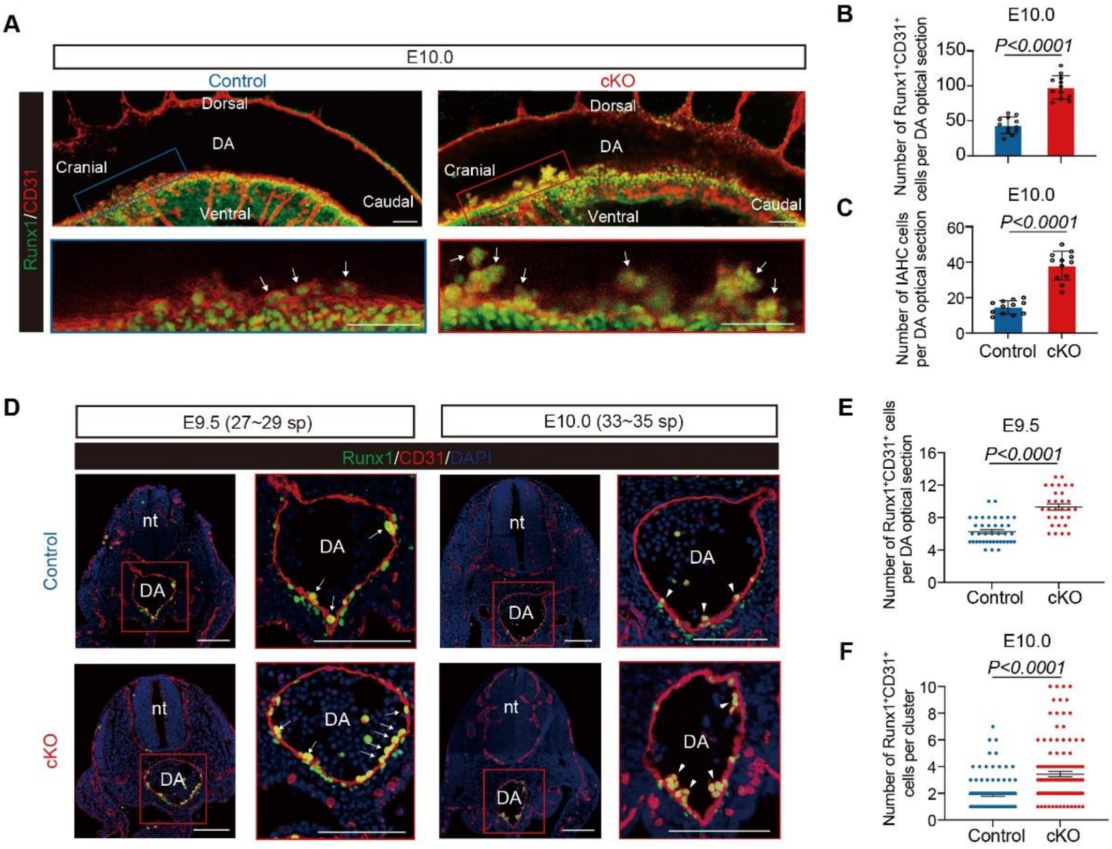

Supplementary Figure 2. Loss of Nupr1 led to an increase in IAHC cells generation in AGM region.

(A) Representative whole-mount confocal images of E10.0 Control and cKO AGM regions stained with CD31 and Runx1 (upper). The boxed floor of the DA regions is shown at higher magnification (lower). Arrows indicate Runx1$^+$CD31$^+$ cells. DA, dorsal aorta. Scale bars, 50 μm.

(B-C) Quantification of Runx1$^+$CD31$^+$ cells (B), and budding IAHC cells with distinct borders (C) on each consecutive optical sections of DAs. 4 Control and 4 cKO embryos were analyzed. Data are represented as mean ± SD and analyzed by unpaired two-tailed Student’s t test.

(D) Representative immunostaining on cross sections at the AGM region of E9.5

(left) and E10.0 (right) Control and cKO embryos. Arrows indicate Runx1$^+$CD31$^+$ cells. Arrowheads indicate hematopoietic cluster. nt, neural tube; DA, dorsal aorta. Scale bars, 100 μm.

(E) Quantification of Runx1$^+$CD31$^+$ cells on each consecutive optical sections of E9.5 (27-29 sp) DA. 5 Control and 3 cKO embryos were analyzed. Data are represented as mean ± SD and analyzed by unpaired two-tailed Student's t test.

(F) Quantification of Runx1$^+$CD31$^+$ cells in individual cluster of E10.0 (33-35 sp) DA. Each dot represents one cluster. 6 Control and 4 cKO embryos were analyzed. Data are represented as mean ± SD and analyzed by unpaired two-tailed Student's t test.

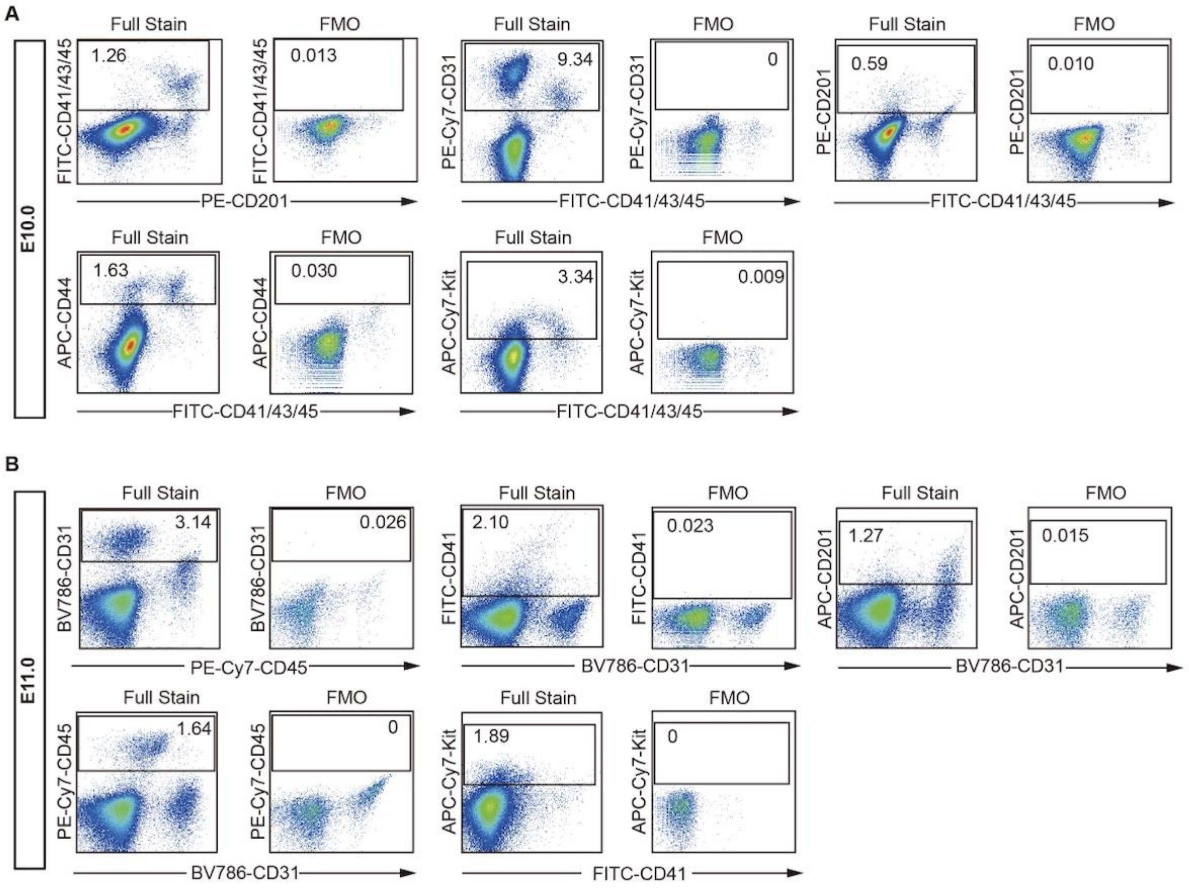

Supplementary Figure 3. Fluorescence Minus One (FMO) controls related to Figure 2.

(A) FMO controls of HECs ($CD41^-CD43^-CD45^-CD31^+CD44^+Kit^+CD201^+$) and IAHC cells ($CD31^+Kit^{high}$) in E10.0 caudal half regions related to Figure 2A.

(B) FMO controls of T1 pre-HSC ($CD31^+CD45^-CD41^{low}Kit^+CD201^{high}$) and T2 pre-HSC ($CD31^+CD45^+Kit^+CD201^{high}$) in E11.0 AGM regions related to Figure 2C.

Supplemental Figure 4

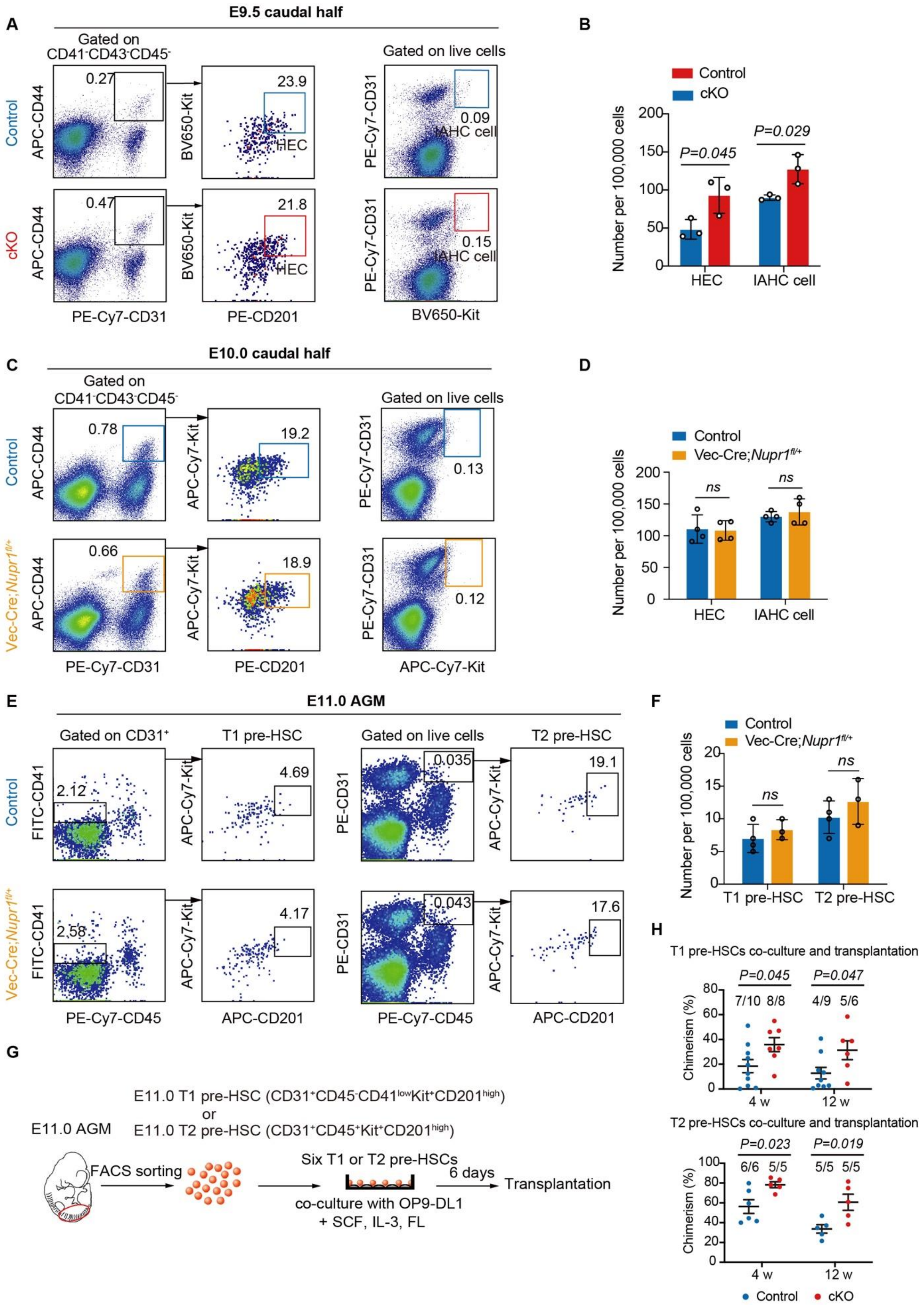

Supplementary Figure 4. Loss of endothelial Nupr1 promoted HEC

specification.

(A) Representative flow cytometric analysis of HEC ($CD41^-CD43^-CD45^-CD31^+CD44^+Kit^+CD201^+$) and IAHC cells ($CD31^+Kit^{high}$) in E9.5 (27-29 sp) Control and cKO caudal half regions.

(B) Graph showing the number of HEC and IAHC cells in (A). Data are collected from 3 independent experiments. Data are represented as mean ± SD and analyzed by unpaired two-tailed Student's t test.

(C) Representative flow cytometric analysis of HEC ($CD41^-CD43^-CD45^-CD31^+CD44^+Kit^+CD201^+$) and IAHC cells ($CD31^+Kit^{high}$) in E10.0 Control and Vec-Cre;*Nupr1*$^{fl/+}$ caudal half regions.

(D) Graph showing the number of HEC and IAHC cells in (C). Data are collected from 3 independent experiments. Data are represented as mean ± SD and analyzed by unpaired two-tailed Student's t test.

(E) Representative flow cytometric analysis of T1 pre-HSC ($CD31^+CD45^-CD41^{low}Kit^+CD201^{high}$) and T2 pre-HSC ($CD31^+CD45^+Kit^+CD201^{high}$) in E11.0 Control and Vec-Cre;*Nupr1*$^{fl/+}$ AGM regions.

(F) Graph showing the number of T1 and T2 pre-HSCs in (E). Data are collected from 3 independent experiments. Data are represented as mean ± SD and analyzed by unpaired two-tailed Student's t test.

(G) Schematic diagram of T1 and T2 pre-HSCs co-culture and transplantation.

(H) Donor chimerism in peripheral blood of recipients after T1 and T2

pre-HSCs co-culture and transplantation monitored at 4 and 12 weeks. The recipients demonstrating ≥5% donor-derived chimerism in peripheral blood were counted as successfully reconstituted. Data are collected from 3 independent experiments. Data are represented as mean ± SEM and analyzed by unpaired two-tailed Student's t test.

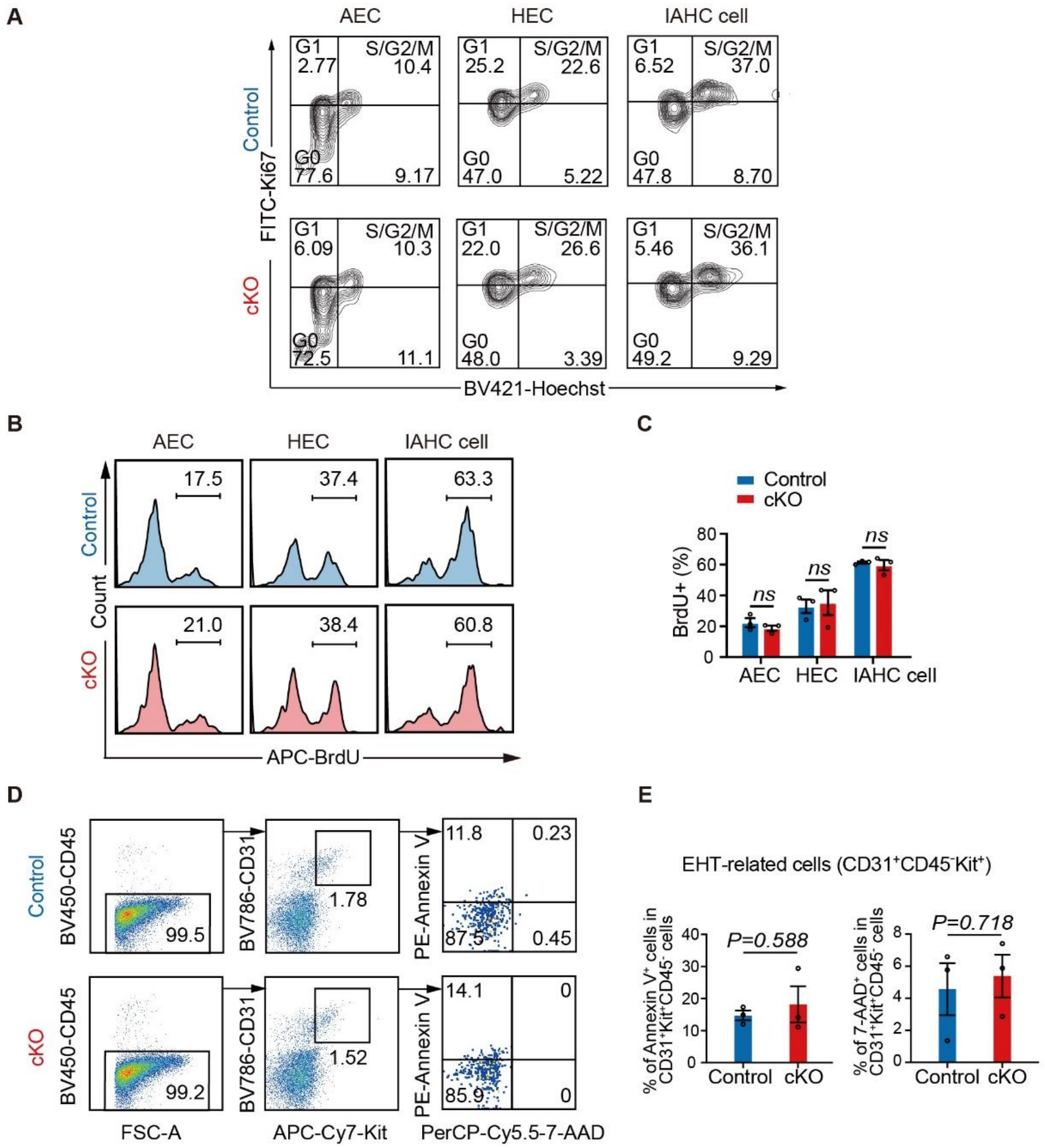

Supplementary Figure 5. Loss of endothelial Nupr1 has no effect on cell cycle status of EHT-related cell populations.

(A) Representative FACS analysis showing the cell cycle status of AECs, HECs, and IAHC cells in E10.0 Control and cKO caudal half regions by Hoechst/Ki67 staining.

(B) Representative FACS analysis showing the cell cycle status of AECs, HECs, and IAHC cells in E10.0 Control and cKO caudal half regions by BrdU

incorporation assay.

(C) Histogram showing the incorporation rates of BrdU in AEC, HEC, and IAHC cells in E10.0 Control and cKO caudal half regions. Data are collected from 3 independent experiments. Data are represented as mean ± SEM and analyzed by unpaired two-tailed Student's t test.

(D) Representative FACS analysis of the cell death status in EHT-related cells ($CD31^+CD45^-Kit^+$) in E10.0 Control and cKO AGM regions by Annexin V/7-AAD staining.

(E) Histograms showing the proportion of Annexin V^+ and 7-AAD^+ cells in (D). Data are collected from 3 independent experiments, and represented as mean ± SEM and analyzed by unpaired two-tailed Student's t test.

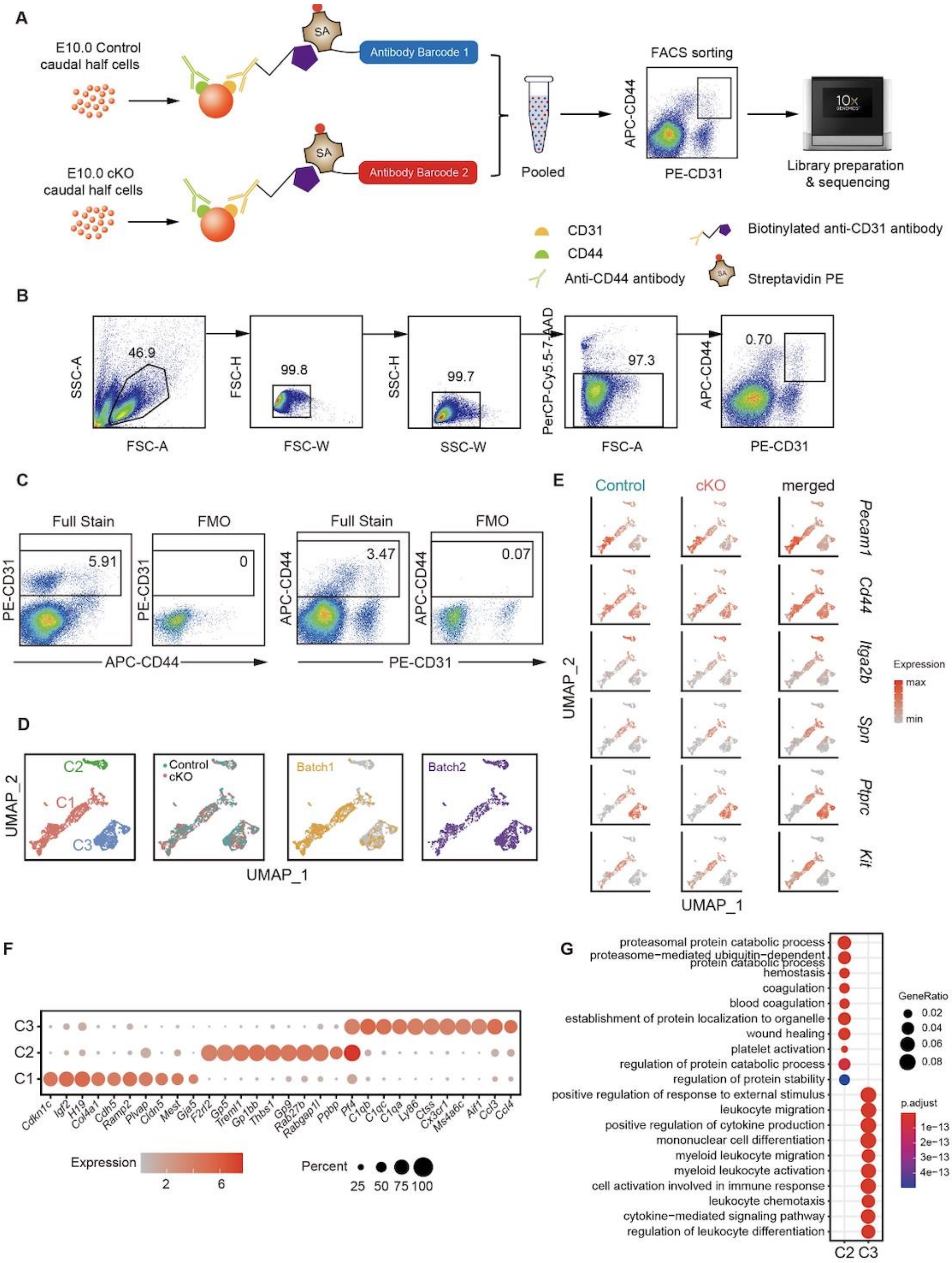

Supplementary Figure 6. Quality control and unsupervised clustering of scRNA-seq.

(A) Schematic overview of samples multiplexing for single-cell RNA sequencing by cell hashing.

(B) Representative FACS analysis depicting sorting strategy for $CD31^{+}CD44^{+}$ cells in E10.0 (32-33 sp) Control and cKO caudal half regions.

(C) FMO controls of $CD31^{+}CD44^{+}$ cells in E10.0 (32-33 sp) Control and cKO caudal half regions related to (B).

(D) UMAP showing the cell clusters of merged or each replicate with the distribution of control and cKO cells.

(E) The expression levels of surface maker genes and Kit for FACS sorting in Control, cKO and merged cells respectively.

(F) Dot plot displaying the expression levels of top 10 DEGs of C1, C2 and C3 cluster.

(G) Dot plot showing the enriched GO terms in C2 and C3.

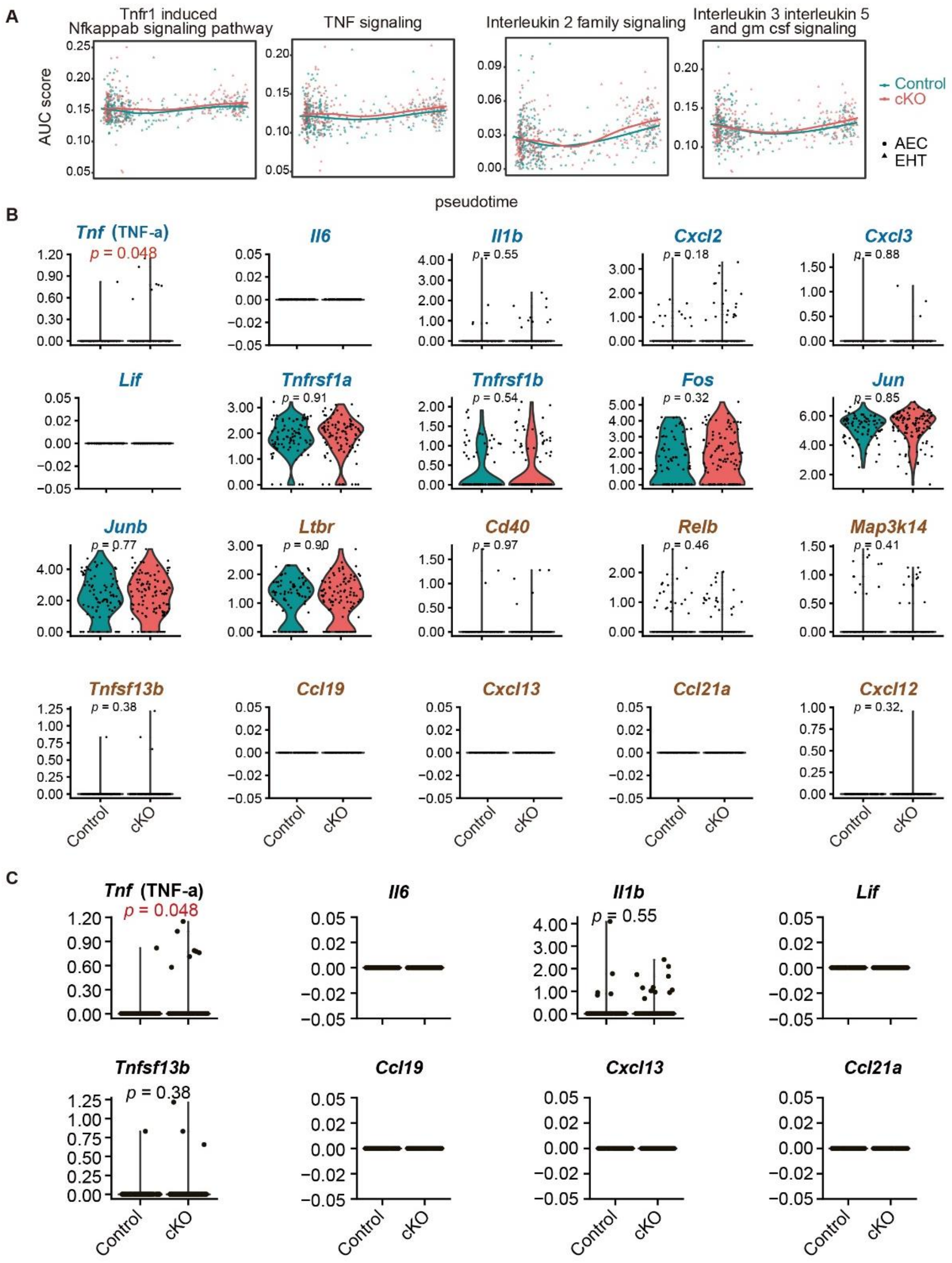

Supplementary Figure 7. Pro-inflammatory genes were upregulated in Nupr1 deficient cells.

(A) Scatter plots displaying the activity score of pathways selected from Enriched Reactome pathways in EHT cells in Figure 5C, Loess smoothed fit curves in Control and cKO embryos were shown along with pseudotime.

Pathway activity score are calculated by AUCell package.

(B) Violin plot show the expression level of signature and downstream target genes of canonical and non-canonical NF-κB signaling pathway. Two-Sample t-Test was used to evaluated the difference between control and cKO embryos. Canonical NF-κB signaling related genes were marked in blue while non-canonical were marked in brown.

(C) Expression level of several pro-inflammatory cytokine genes in Control and cKO cells.
